# Widely targeted metabolomics and SPME-GC-MS analysis revealed the quality characteristics of non-volatile/volatile compounds in Zheng’an Bai tea

**DOI:** 10.3389/fnut.2024.1484257

**Published:** 2024-11-25

**Authors:** Li Liu, Dahe Qiao, Xiaozeng Mi, Shirui Yu, Tingting Jing, Yanlin An

**Affiliations:** ^1^Department of Food Science and Engineering, Moutai Institute, Renhuai, China; ^2^Guizhou Tea Research Institute, Guizhou Academy of Agricultural Sciences, Guiyang, China; ^3^State Key Laboratory of Tea Plant Biology and Utilization, Anhui Agricultural University, Hefei, China

**Keywords:** Zheng’an Bai tea, widely targeted metabolomics, non-volatile/volatiles, fresh leaves, tea processing, SPME-GC-MS

## Abstract

**Background:**

As albino tea under the geographical protection of agricultural products, Zheng’an Bai tea is not only rich in amino acids, polyphenols and other beneficial components for the human body, but also its leaf color will turn green as the temperature gradually rises, thus causing changes in the quality characteristics of tea leaves. However, these changing characteristics have not yet been revealed.

**Methods:**

In-depth quality analysis was carried out on the fresh leaves of Zheng’an Bai tea at four different developmental stages and four samples from the processing stage through extensive targeted metabolomics and SPME-GC-MS analysis.

**Results:**

In this study, a total of 573 non-volatile metabolites were detected from the fresh leaves and processing samples of Zheng’an Bai tea, mainly including 96 flavonoids, 75 amino acids, 56 sugars and alcohols, 48 terpenoids, 46 organic acids, 44 alkaloids, and 39 polyphenols and their derivatives. In fresh leaves, the most significant differential metabolites (VIP > 1, *p* < 0.05) among different samples mainly include substances such as ethyl gallate, theaflavin, isovitexin and linalool, while the main differential metabolites of samples in the processing stage include alkaloids, polyphenols and flavonoids such as zarzissine, methyl L-Pyroglutamate, theaflavin 3,3’-digallate, euscaphic acid and ethyl gallate. Overall, substances such as sugars and alcohols, alkaloids and polyphenols show the greatest differences between fresh leaves and the processing process. Meanwhile, 97 kinds of volatile metabolites were detected in these samples, most of which had a higher content in the fresh leaves. Moderate spreading is conducive to the release of the aroma of tea leaves, but fixation causes a sharp decrease in the content of most volatile metabolites. Ultimately, 9 volatile substances including geraniol, linalool, nerolidol, jasmone, octanal, 1-Nonanal, heptaldehyde, methyl salicylate and 1-Octen-3-ol were identified as the key aroma components (OAV >1) of Zheng’an Bai tea.

**Conclusion:**

In conclusion, this study has for the first time comprehensively revealed the quality change characteristics of fresh leaves at different developmental stages and during the processing of Zheng’an Bai tea, and provided a foundation for further process improvement.

## 1 Introduction

The unique taste, pleasant aroma, and abundant nutrients have made tea one of the most popular non-alcoholic beverages (1). The widespread cultivation of tea plants [*Camellia sinensis* (L.) O. Kuntze] worldwide has made them one of the most important cash crops for many countries to increase farmers’ income. China is the origin country of tea and has abundant tea tree resources, hundreds or even thousands of tea varieties are made into six traditional tea types such as green tea and black tea with different flavors according to their quality characteristics and different processing procedures (2, 3). Meanwhile, in recent years, some naturally mutated tea plant resources have attracted increasing attention from researchers due to their characteristics such as low caffeine or high anthocyanins (4, 5). “Zheng’an Bai tea” is a famous green tea produced in Zheng’an County, Guizhou Province, made from the tender buds and leaves of the naturally mutated tea cultivar “Baiye 1.” Due to its high amino acid content and other characteristics, it has been listed as a Chinese National Geographical Indication Product since 2011 (6). Unlike other yellowing or purpling tea plant varieties, “Zheng’an Bai tea” belongs to the temperature-sensitive variety, whose leaves appear white at lower temperatures and gradually turn green as the temperature rises. Although many reports have conducted in-depth studies on the molecular regulatory basis of albino tea plants, the quality of tea is influenced by many factors such as climate, water and fertilizer, and processing (7–9). At present, there are no reports on the metabolic profiles of different leaf positions and the quality change characteristics of fresh leaves during processing.

Compared with common tea varieties, naturally mutated tea varieties often have unique quality characteristics. For example, 22 anthocyanins were identified in purple tea varieties such as “Zijuan” or “Ziyan,” and their contents reached more than 1 μg/g (dry weight) (10); while in the yellow-leaf tea variety, the theanine content of “Zhonghuang 2 (yellow-leaf tea)” was significantly higher than that of “Longjing 43 (normal tea)” (11). For tea varieties with albino leaves, both light-sensitive and temperature-sensitive varieties have the characteristics of low polyphenol content and high amino acid content, which gives the tea soup a higher freshness (12). Many studies have shown that with the development of tea leaves, the secondary metabolite profiles such as catechins, caffeine, theanine, and anthocyanins will undergo significant changes (13). For “Zheng’an Bai tea,” the amino acid content gradually decreases with the development of the leaves, the contents of catechin (EGC), epicatechin gallate (ECG), epicatechin (EC), catechin (C), epigallocatechin gallate (EGCG), and gallocatechin (GC) in albino or yellowing variegated leaves were significantly lower than those in normal leaves (14). But due to factors such as altitude and unique climate, the metabolic profile characteristics of different leaf positions still need to be revealed.

Although volatile components account for only 0.01% of the dry weight of tea, they contribute to the main aroma quality of tea (15, 16). Many studies have conducted in-depth analysis of the volatile metabolites and aroma changing characteristics in tea. For example, Xia et al. (17) analyzed the influence of three fixation methods on the aroma quality of “Anji Bai tea” (albino tea), identified 9 key components that caused the aroma changes of Anji Bai tea, and proved that linalool and geraniol contribute to the formation of floral, fruity and honey aromas of tea; the analysis of harvest seasons and etiolated varieties revealed that the relative content of volatile compounds in steamed green tea was significantly negatively correlated with the season (*p* < 0.05). The contents of volatile compounds such as (+)-δ-cadinene, farnesyl acetone, carvone, trans-β-ionone and nerolidol were higher in spring tea. However, the differences in the total volatile compounds among the three albino varieties of steamed green tea were not significant (*p* > 0.05) (18). Compared with “Yinghong 9,” the “Huangyu” variety contains higher levels of α-farnesene, β-cyclocitral, nerolidol and trans-geranylacetone, which have been confirmed to be related to the flower and fruit aroma in the fermented leaves (19). It was found in the study of purple tea that anaerobic treatment facilitated the accumulation of 2-heptanol, (E)-2-hexenal, ethyl salicylate, phenylethyl alcohol and (E, E)-2, 4-decadienal, but inhibited the formation of (Z)-3-hexenyl acetate and methyl jasmonate (20). Furthermore, Gao et al. also detected and analyzed the volatile substances in the flowers of three albino tea plants and one normal tea plant, and discovered that acetophenone and (R)-1-phenylethanol were positively correlated with the sweet flavor, while methyl salicylate, 2-heptanol, (E)-2-hexenal, nonanal and 2-pentanol were positively correlated with the green smell (21). The above studies mainly focused on the change characteristics of volatile metabolites in specific process conditions or tissues, and there were no studies on the change trend of volatile metabolites/aroma in tea leaves at different development stages and during the whole processing process. Therefore, the revelation of aroma changes of “Zheng’an Bai tea” still needs to be strengthened (22–24).

In this study, we focused on exploring the dynamic changes of non-volatile/volatile metabolites throughout the entire process from the fresh leaves, the processing, to the finished tea of “Zheng’an Bai tea.” A total of eight stages of samples, including buds (BUD), the first leaf (FL), the second leaf (SL), the first bud and the first leaf (FBFL), spreading for 3 h (TF3h), spreading for 6 h (TF6h), fixation (SQ) and drying (DRY), were detected and analyzed by widely targeted metabolomics and GC–MS techniques to comprehensively reveal the metabolic profile and key aroma components of “Zheng’an Bai tea.” This will help us recognize and understand the formation basis of the quality of “Zheng’an Bai tea” and the influence brought by the processing techniques.

## 2 Materials and methods

### 2.1 Plant materials and reagents

The samples of buds (BUD), the first leaf (FL), the second leaf (SL), the first bud and the first leaf (FBFL) of “Baiye 1” and samples at different processing stages (spreading for 3 h, TF3h; spreading for 6 h, TF6h; fixation, SQ; drying, DRY) were collected. All these samples were collected from the tea garden of Zhongguan Town, Zheng’an County, Guizhou Province (28°43′ N, 107°61′ E). Some of the collected fresh samples were transported to the laboratory in dry ice and stored in a − 80°C refrigerator for later use, while the other portion was processed into dry tea according to the following process: The first buds and first leaf collected was placed in a well-ventilated room at room temperature for 6 h of spreading. The tea was turned over 2–3 times during spreading to maintain a humidity level between 75 and 85%. After spreading, the tea was quickly straightened in a tea straightening machine and then fixed at 230°C for 4–5 min. Finally, it was dried at 110°C for 1.5 h in a dryer. Deionized water was produced using a Milli-Q water purification system (Millipore, Billerica, Massachusetts). Methanol, acetonitrile, and ammonium acetate (LC–MS grade) were obtained from Merck (Darmstadt, Germany), and formic acid was obtained from TCI (TCI America). All the samples were stored in a − 80°C refrigerator until they were detected.

### 2.2 Sample preparation and extraction

Before the samples were formally prepared, all the tea leaves were freeze—dried for 24 h first to ensure the consistency of water content. Then, weigh 50 mg of the freeze-dried sample and add 1 ml of the extraction solution (methanol:acetonitrile:water volume ratio = 2:2:1). Vortex the centrifuge tube containing the sample for 30 s to ensure sufficient mixing. Then, add steel balls, perform ultrasonic treatment at 45 Hz for 10 min, followed by low temperature ultrasonic treatment for 10 min, and then stand at −20°C for 1 h (25, 26). After standing, the sample was centrifuged at 12,000 rpm for 15 min at 4°C, and 500 μl of the supernatant was taken and dried in a vacuum concentrator. Add 160 μl of extraction solution (acetonitrile:water volume ratio 1:1) to dissolve the metabolites; vortex for 30 s, and ultrasonicate for 10 min in an ice-water bath; centrifuge the sample at 12,000 rpm for 15 min at 4°C; carefully remove 120 μl of the supernatant into a 2 ml injection bottles for testing. All samples included three replicates.

### 2.3 UPLC-ESI-MS/MS analysis

A Waters UPLC Acquity I-Class PLUS ultra-high performance liquid chromatography was coupled to an AB Sciex Qtrap 6,500+ mass spectrometer system for the detection of metabolites (UPLC-ESI-MS/MS). The specific UPLC conditions are as follows: C18 column (1.8 μm, 2.1 mm × 100 mm, Acquity UPLC HSS T3), mobile phase A is a mixture of 0.1% formic acid and 5 mM ammonium acetate aqueous solution, and mobile phase B is 0.1% formic acid acetonitrile. The flow rate is 350 μl/min. The gradient elution conditions are: 98:2 v/v at 0 min, hold for 1.5 min, 50:50 v/v at 5 min, 2:98 v/v at 9 min, hold for 1 min; 98:2 v/v at 11 min, hold for 3 min, and the injection volume is 2 μl. The temperature of the electrospray ionization (ESI) source was set at 550°C, and the ion source gases I (GSI), gas II (GSII), and curtain gas (CUR) were set at 50, 55, and 35 psi, respectively. The collision-induced ionization parameters were set to medium. For more detailed methods, please refer to the research of Shi et al. (27). Based on the GB-PLANT commercial database, qualitative/quantitative mass spectrometry analysis was performed on the metabolites of the samples. The characteristic ions of each substance were screened out by triple quadrupole, and the signal intensity of the characteristic ions was obtained in the detector. After obtaining the metabolite mass spectrometry analysis data of different samples, the peak area integration was performed on all the mass spectrometry peaks, and the integration correction was performed on the mass spectrometry peaks of the same metabolite in different samples. Based on KEGG databases, the identified metabolites were classified and pathway analyzed.^1^

### 2.4 Extraction of volatile compounds

The volatile substances in tea were enriched by the method of automatic solid-phase microextraction (SPME) (28). After freeze-drying the tea samples, use a ball mill to grind the freeze-dried tea samples into powder, take 0.100 g and place it in a 15 ml gas chromatography–mass spectrometry glass bottle, and add 5 μl of ethyl decanoate at 1 μg/ml as an internal standard. Adsorption was conducted using the SPME Arrow of model 36SP05T3 (C-WR/PDMS 80/10-P3) from Thermo Scientific, and its adsorption phase was mainly PDMS (Polydimethylsiloxane). Place the sample bottle at 60°C, adsorb for 50 min, and then use GC–MS to detect volatile substances. GC conditions: Inlet temperature of 250°C, thermal desorption of volatile components for 10 min, separation using a fused silica chromatographic column (DB-5, 30 m × 0.25 mm × 0.25 μm, Folsom, USA). Carrier gas: Helium at a flow rate of 1 ml/ min; the starting temperature of the column oven is 50°C, maintained for 2 min, raised to 80°C at a rate of 2°C/min, maintained for 1 min; raised to 100°C at a rate of 3°C/min, maintained for 4 min; raised to 130°C at a rate of 3°C/min, maintained for 4 min; raised to 150°C at a rate of 5°C/min, maintained for 0 min; raised to 200°C at a rate of 10°C/ min, maintained for 0 min; raised to 240°C at a rate of 20°C/min, maintained for 3 min. Mass spectrometry conditions: Ion source EI, electron energy 80 eV, full ion scan mode, mass scan range 41–350 m/z. Finally, the volatile metabolites were identified based on the National Institute of Standards and Technology (NIST) mass spectrometry database and retention index (RI) (24, 29, 30).

### 2.5 Semi-quantitation and calculation of odor activity values

The concentration of volatile compounds was calculated based on their peak areas and the peak area of the internal standard compound. The odor activity value (OAV) of the volatiles was obtained by dividing the concentration by their odor threshold (15, 31–37). Furthermore, some flavor thresholds were obtained through the online data of VCF (Volatile Compounds in Food; https://www.vcf-online.nl/VcfHome.cfm). In addition, it should be noted that all metabolite detections were entrusted to Biomarker Technologies Co., Ltd.

### 2.6 Statistics analysis

The original peak area information of each substance is normalized according to the total peak area of the sample (38). And PCA analysis and Spearman correlation analysis were performed to verify the reproducibility of the intra-group samples and control samples. Based on the grouping information, the fold change of metabolites between different groups was calculated and compared, and the significance *p*-value of each compound was calculated using the T-test. At the same time, the (O)PLS-DA model was constructed based on the online analysis toolbox BMKCloud.^2^ The screening criteria for different metabolites were *p* < 0.05, and VIP > 1 (39). In all (O)PLS-DA analyses, the values of R2Y and Q2 of the model are both greater than 0.9.

## 3 Results and discussion

### 3.1 Overall view of non-volatile metabolites in Zheng’an Bai tea

**Figure 1:**
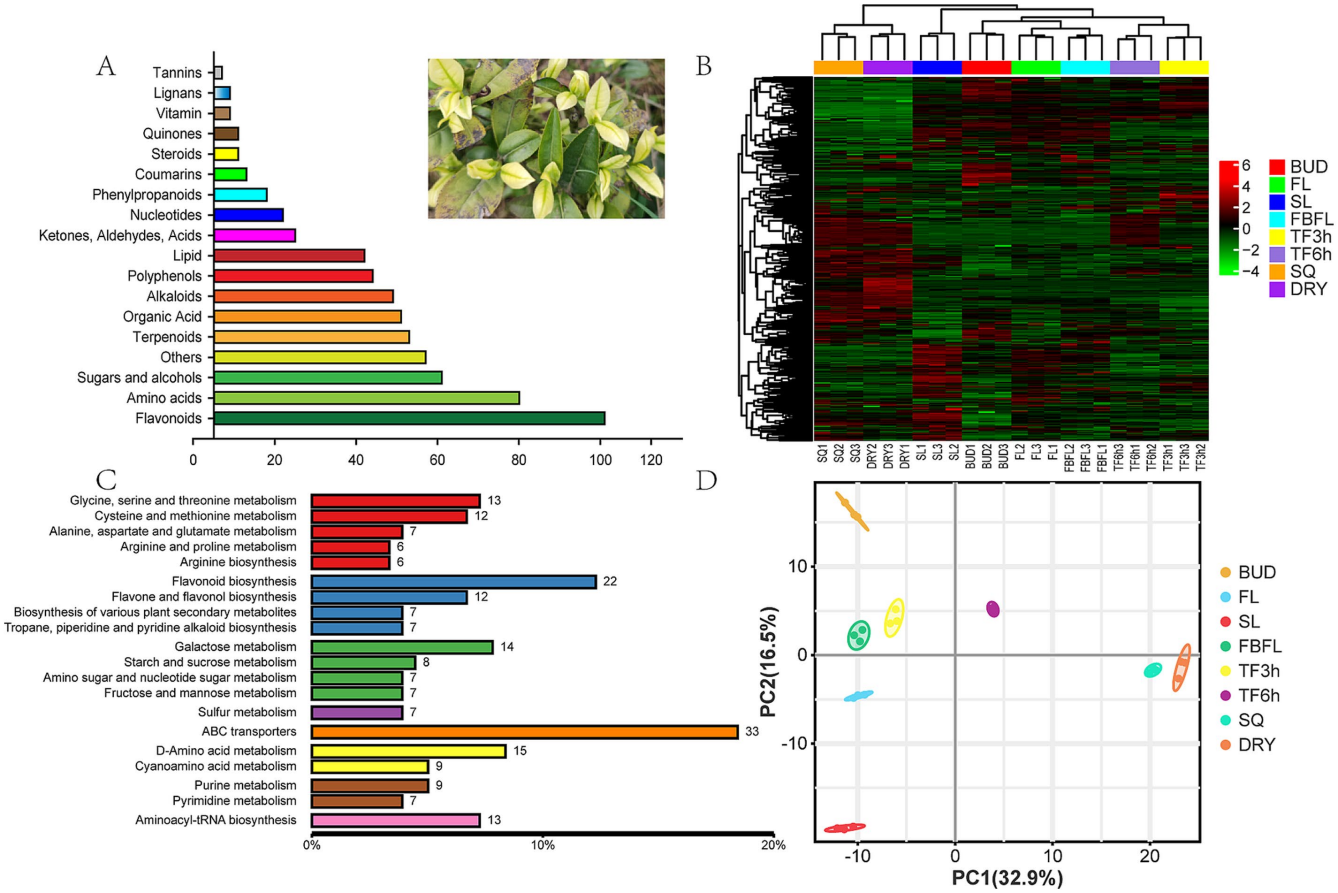
Overview of non-volatile metabolites. **(A)** Classification of non-volatile metabolites, **(B)** heatmap analysis of non-volatile metabolites, **(C)** the main enrichment pathway for non-volatile metabolites, **(D)** PCA analysis based on non-volatile metabolites.

A total of 573 non-volatile metabolites were detected in fresh leaves at different development stages and samples at different processing stages of “Zheng’an Bai tea,” including 96 flavonoids and their derivatives, 75 amino acids and their derivatives, 56 saccharides and alcohols, 48 terpenoids and their derivatives, 46 organic acids, 44 alkaloids and their derivatives, 39 polyphenols and their derivatives, 13 phenylpropanoids and 12 lignans and coumarins (Figure 1A). While Wang et al. (40) identified a total of 527 non-volatile metabolites in green tea, including 109 flavonoids, 89 phenolic acids, 81 lipids, 64 amino acids and their derivatives, 37 organic acids, 25 alkaloids, and 12 sugars and alcohols. In comparison, Zheng’an Bai tea contains fewer flavonoids and a greater variety of amino acids. Meanwhile, Spearman Rank Correlation analysis showed that there was good biological reproducibility among the samples within each group, which indicated that the data in this study had good reproducibility and reliability (Supplementary Figure S1).

Metabolites interact with each other in organisms to form different pathways. By annotating all identified metabolites through the KEGG database, the top 20 annotation information indicates that a large number of secondary metabolites such as amino acids and flavonoids are enriched, reflecting the abundant content of these substances in “Zheng’an Bai tea” (Figures 1B,C). Based on the metabolomics data of 573 non-volatile metabolites (Supplementary Table S1), PCA analysis was performed using an unsupervised pattern. The results showed that these samples were clearly classified into 9 different groups, suggesting that there may be significant differences in non-volatile metabolism between different groups (Figure 1D). The first principal component and the second principal component explained 32.9 and 16.5% of the variation results, respectively. Compared with other samples, the fixing and drying samples were closely clustered together, indicating that the metabolic differences between these samples were relatively small.

### 3.2 The content changes of non-volatile metabolites in different tissues

**Figure 2:**
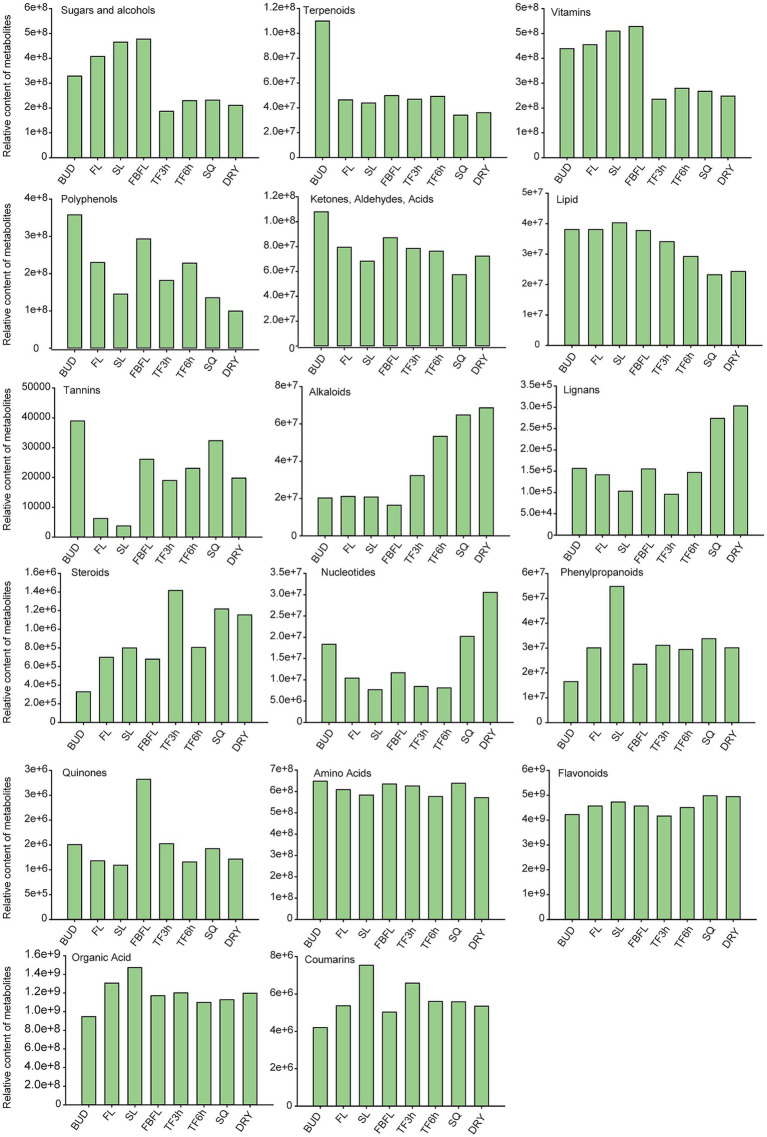
The changing trends of different types of non-volatile metabolites in eight tissues (the relative contents were calculated based on the peak areas of metabolites in the widely targeted metabolomics).

Polyphenols are one of the most important components of tea tree leaves (41). Overall, the buds of “Zhengan Bai tea” contain a relatively large amount of polyphenols and terpenoids, and ultimately show a lower content in the dried samples (Figure 2). The contents of sugars, alcohols and vitamins in tea leaves after spreading are all lower than those in fresh leaves. In addition, the lipid content gradually decreases during the processing process, which is similar to the research results of Li et al. (42). While substances such as alkaloids, lignins and nucleotides generally showed an increasing trend during the processing. Amino acids, organic acids and flavonoids are important taste substances and beneficial components in tea (27, 43). Their contents change slightly in fresh leaves at different stages and during the processing. Furthermore, Figure 2 also shows the dynamic change characteristics of substances such as tannins, ketones, aldehydes, acids and coumarins in different tissues. Compared with other stages, the high temperature in the fixation stage leads to the most drastic changes in metabolites, which is consistent with the previous research results (17, 40).

### 3.3 The non-volatile differential metabolites among the fresh leaves

**Figure 3:**
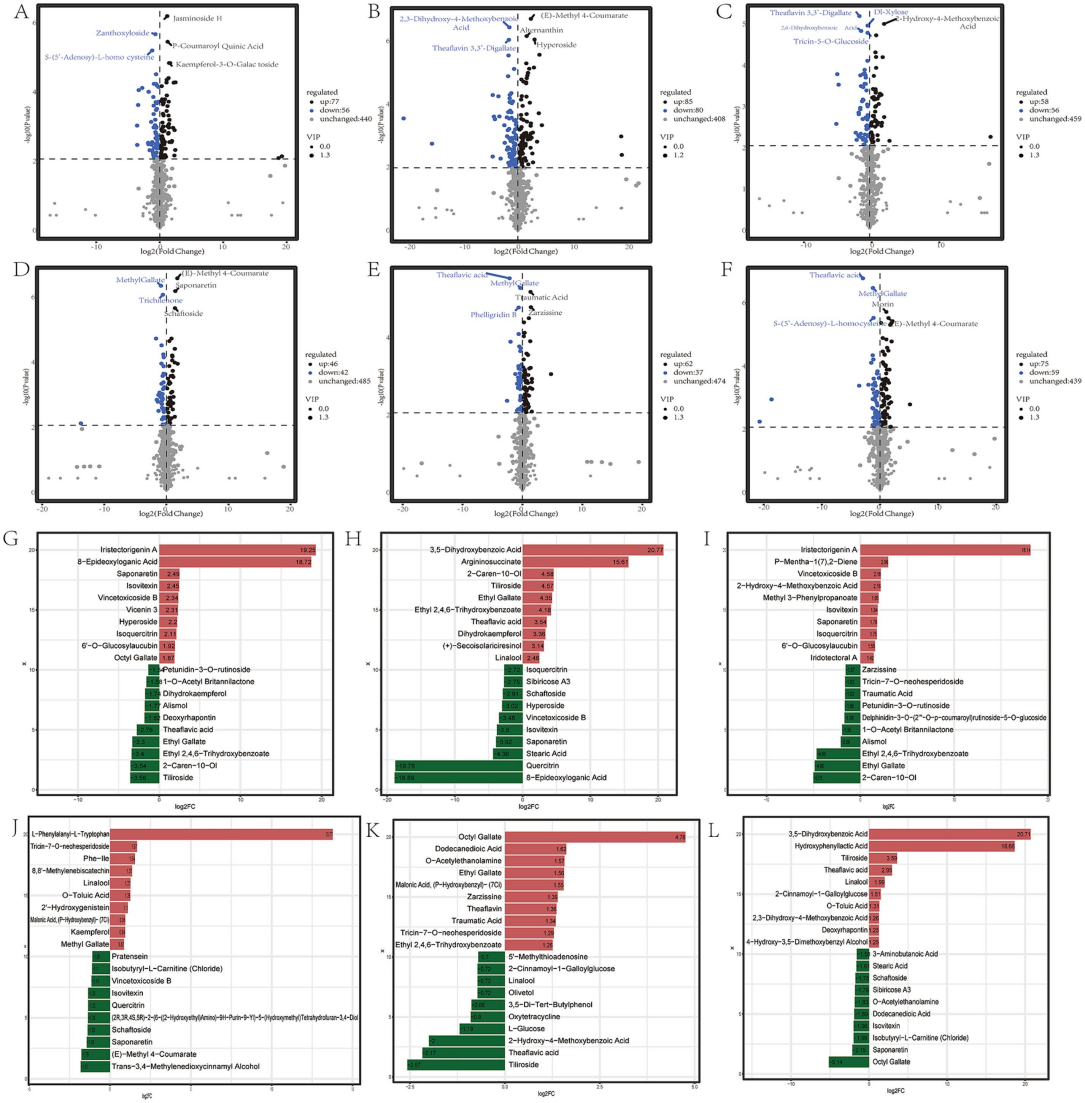
Differential metabolites of different fresh leaf samples. **(A–F)** Represent the number of differential metabolites in BUD vs. FL; BUD vs. SL; BUD vs. FBFL; FL vs. SL; FL vs. FBFL, and SL vs. FBFL, respectively; **(G–L)**, respectively, represent the top 20 metabolites with the largest fold change in the comparison groups of BUD vs. FL; BUD vs. SL; BUD vs. FBFL; FL vs. SL; FL vs. FBFL, and SL vs. FBFL.

Many studies have shown that the quality characteristics of different leaf positions of tea plants are significantly different (1, 44, 45). For Zheng’an Bai tea, 133,165,114 differential metabolites were detected in the FL (first leaf), SL (second leaf) and FBFL (first bud and first leaf) compared to the bud, respectively (Figures 3A–C). A total of 88 and 99 differential metabolites were identified between the first and second leaves and first buds and first leaf, respectively. At the same time, a total of 114 differential metabolites were found between the FBFL and the SL (Figures 3D–F). For fresh leaves at different stages of development, the metabolites with top 20 fold changes values contain a large number of sugars, alcohols, organic acids, amino acids, polyphenols, flavonoids and alkaloids (Figures 3G–L; Supplementary Figures S2A–F). In addition, many terpenes, alkaloids and polyphenols also showed diverse variation characteristics at different leaf positions. Further KEGG enrichment analysis showed that flavonoids and flavonoid biosynthesis pathways were enriched in fresh leaves of tea plants at different developmental stages (Supplementary Figures S2G–L). Among the top 20 metabolites with the largest fold changes, ethyl gallate, theaflavic acid, isovitexin, linalool and vincetoxicoside B were the most common. All the differential metabolites and OPLS—DA model information are presented in Supplementary Table S2 and Supplementary Figure S3, respectively.

### 3.4 Characteristics of the quality change of non-volatiles in the processing process

**Figure 4:**
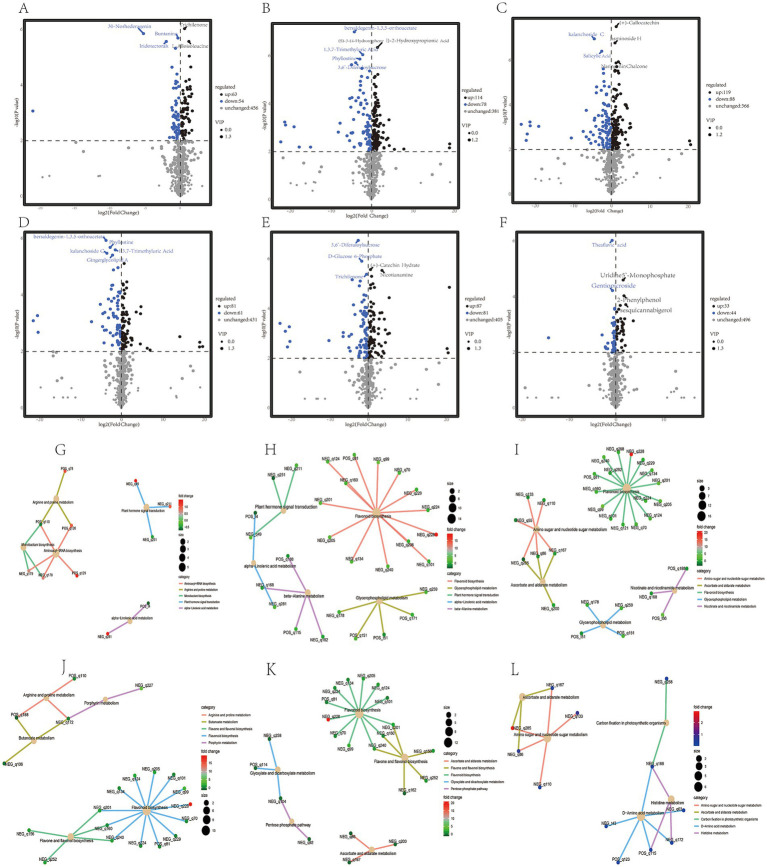
Differential metabolite heat map analysis and KEGG enrichment analysis of different samples during processing. **(A–F)** Represent the number of differential metabolites in TF3h vs. TF6h; TF3h vs. SQ; TF3h vs. DRY; TF6h vs. SQ; TF6h vs. DRY, and SQ vs. DRY, respectively. (**G–L)** Represent the KEGG enrichment analysis of the differential metabolites in TF3h vs. TF6h; TF3h vs. SQ; TF3h vs. DRY; TF6h vs. SQ; TF6h vs. DRY, and SQ vs. DRY, respectively.

The quality of tea beverages is not only related to fresh leaves, but also the processing technology can also significantly affect the quality of dry tea (31, 46). At different stages of processing, the content of many non-volatile metabolites in Zheng’an Bai tea changed significantly. Compared with the TF3h, 117, 192, and 207 different metabolites were identified in the TF6h, SQ, and DRY stages, respectively (Figures 4A–C; Supplementary Figures S3A–C). Compared with TF6h stage, SQ and DRY identified 142 and 168 different metabolites, respectively. However, after fixation, enzymatic reactions in tea decreased significantly, and only 77 different metabolites were identified compared to the DRY tea (Figures 4D–F; Supplementary Figures S4D–F) (47). The above results indicate that the fixation and drying processes are very important for the formation of tea quality (9, 38). Among these different metabolites, there are a large number of flavonoids, polyphenols, terpenoids, sugar alcohols, and organic acids (Supplementary Figures S3G–L). Similar to the research results of Chen et al. in large-leaf black tea (30), during specific processing stages, some alkaloids such as zarzissine, methyl L-Pyroglutamate, piperidine, flavonoids such as theaflavin 3,3′-digallate, terpenoids such as euscaphic acid, and polyphenols such as ethyl gallate changed significantly. KEGG enrichment analysis showed that during different spreading stages, many plant hormones such as salicylic acid and a large amount of amino acids such as arginine were enriched. The different metabolites between the spreading samples (including TF3h and TF6h) and the SQ and DRY samples are all significantly enriched in the flavonoid metabolic pathway. Previous studies have shown that the spreading process can promote the hydrolysis of some proteins to increase the content of amino acids, accompanied by a decrease in the content of tea polyphenols, soluble sugars, and others (40, 48). While the different metabolites between the SQ and DRY samples are mainly enriched in the amino sugar and amino acid pathways (Figures 4G–L).

### 3.5 Total volatiles metabolites in Zheng’an Bai tea

**Figure 5:**
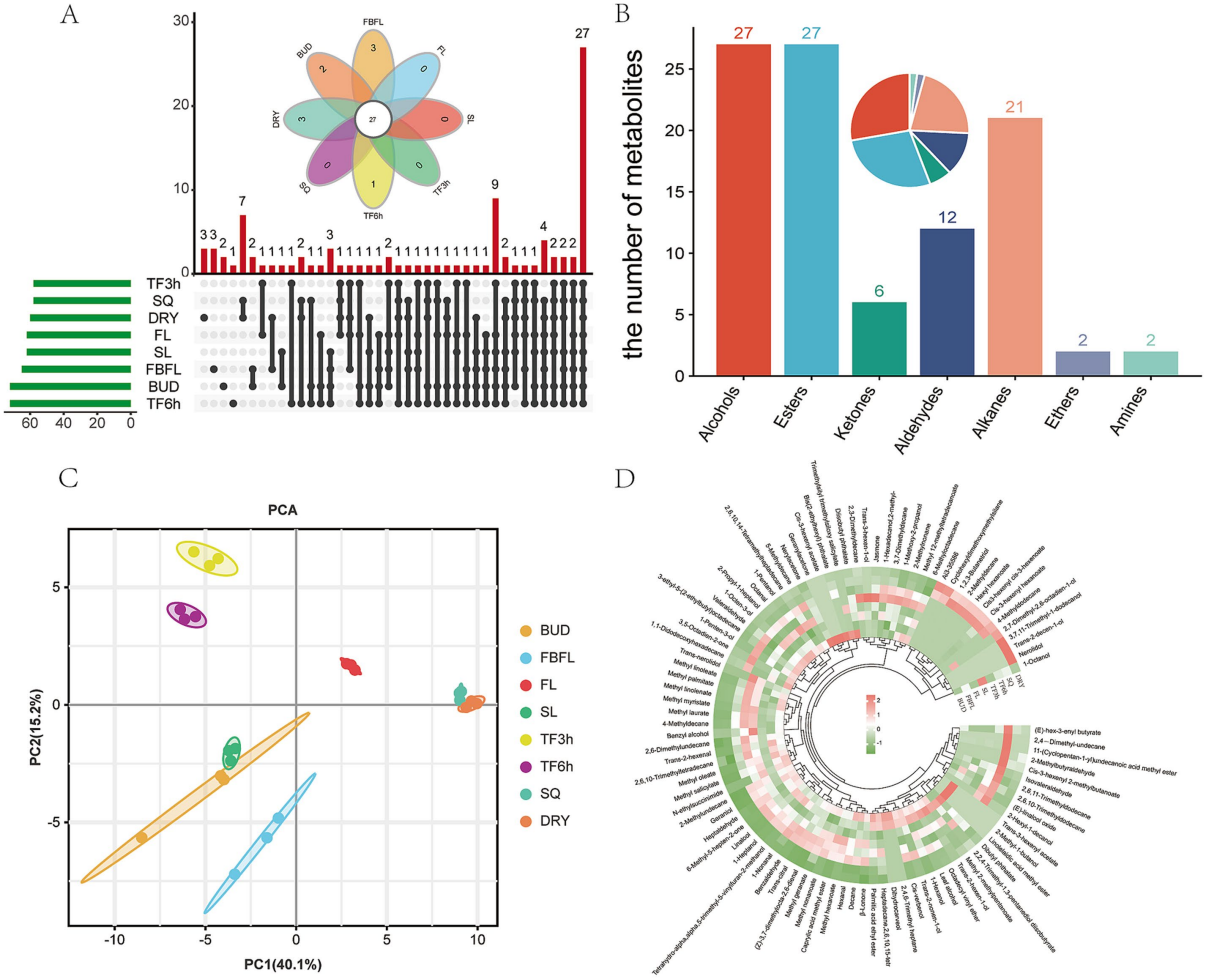
Overview of volatile metabolites. **(A)** Venn diagram analysis of volatile metabolites in different samples, **(B)** Classification statistics of volatile metabolites, **(C)** PCA analysis of volatile metabolites, **(D)** heatmap analysis of volatile metabolites.

A total of 97 volatiles components were detected in the samples of different developing tissues and processing stages of Zheng’an Bai tea (Supplementary Table S3). This number is more than the 47 in Longjing tea and the 91 in Fudingdabai tea (35, 49). However, this may be because leaves at different developmental stages are included in this study. Among them, the number of aroma components detected in the samples of BUD and TF6h was the highest, both of which were 72, while the number of aroma components in the samples of TF3h and SQ was the least, which was 58 (Figure 5A). This once again proves that moderate spreading is beneficial for the release of tea aroma (35). Among the 97 volatile components in these samples, benzyl alcohol, geraniol, nonanal, methyl salicylate, cis-jasmone, hexanal, nonanal and linalool oxide are common characteristic volatile substances in tea (30, 50). All volatiles substances are classified into 7 categories, among which alcohols and esters have the largest number, both being 27, followed by alkanes and aldehydes, with 21 and 12, respectively, (Figure 5B). This result is slightly different from the study of black tea by Yin et al. (51). Similar to the results of the widely-target metabolome analysis, all samples were well divided into eight groups by PCA analysis based on volatiles substances, and the interpretation rates of the first principal component and the second principal component reached 40.1 and 15.2%, respectively (Figure 5C). Meanwhile, similar to the research results of Wang et al. (40), most of the volatiles substances decreased sharply in the fixing stage (Figure 5D).

### 3.6 Key aroma compounds identified using OAVs in the Zheng’an Bai tea

Aroma is one of the important flavor characteristics of tea, which is not only related to the quality of fresh leaves, but also affected by processing technology (2). The odor activity value (OAV) is an important index for objectively evaluating the contribution of volatile substances to aroma and is widely used in many studies (52, 53). Volatile components with an OAV value greater than 1 are usually considered to have an important contribution to the aroma (47). For example, Qin et al. (29) identified 11 OAV > 1 key aroma compounds in steamed green tea through OAV analysis. In this study, we conducted OAV analysis on all volatile components based on the odor threshold values (Supplementary Table S4). As shown in Table 1, the volatile metabolites with an OAV value greater than 1 in at least one tissue are presented in detail. The results indicate that the contents of most volatile metabolites are relatively high in fresh leaves and during the spreading stage, but they decrease sharply or are difficult to be detected after fixation. Nine key aroma components of OAV >1 were identified in the dried tea samples, including 1-Octen-3-ol, nerolidol, linalool, methyl salicylate, jasmone, geraniol, heptaldehyde, 1-Nonanal, and octanal. Among them, 1-octen-3-ol, linalool, methyl salicylate, nonanal and geraniol have odors such as fresh, floral and sweet scents, and they have also been identified as the key aroma substances of Longjing tea (49, 53). While jasmone and octanal are, respectively, one of the key aroma components of Lu′an Guapian tea and Xinyang Maojian tea (28, 29). Meanwhile, heptaldehyde is regarded as one of the contributing components of the chestnut-like aroma in tea (47).

**Table 1:** The OAV value of volatile metabolites (OAV value is greater than 1 in at least one tissues, more detailed information has been shown in Supplementary Table S4).

CAS	Name	Odor description	Threshold (mg/kg)	OAV							
				BUD	FBFL	FL	SL	TF3h	TF6h	SQ	DRY
106-26-3	Neral	Green, grassy, fresh	0.053	13.0	8.6	8.6	10.3	17.1	15.8	0.0	0.0
141-27-5	Trans-citral	Citrus, lemon-like	0.04	2.2	1.5	1.8	1.3	3.1	3.0	0.0	0.0
39028-58-5	(E)-linalool oxide	Floral, honey-like	3	1.0	1.6	0.9	0.2	3.3	3.9	0.2	0.2
3391-86-4	1-Octen-3-ol	Green, vegetative-like	0.007	9.6	6.2	0.0	9.5	0.0	6.6	1.8	1.4
96-17-3	2-Methylbutyraldehyde	Almond, chocolate	0.04	0.0	0.0	0.0	0.0	0.0	3.8	0.2	0.9
30086-02-3	3,5-Octadien-2-one	Creamy and fruity smell	0.0005	725.9	0.0	376.7	0.0	0.0	1026.4	0.0	0.0
544-12-7	Trans-3-hexen-1-ol	Green, leafy, grassy	0.07	0.0	0.0	0.5	0.0	3.9	0.0	0.0	0.0
79-77-6	β-ionone	Floral, sweet	0.09	1.9	1.3	1.1	2.2	1.4	2.3	0.6	0.3
100-51-6	Benzyl alcohol	Floral, rose-like, phenolic	0.1	47.4	0.7	12.8	108.6	44.5	17.0	0.2	0.3
6728-26-3	Trans-2-hexenal	Green, fruity	0.04	3.3	5.1	1.6	2.6	4.9	1.9	0.0	0.0
34995-77-2	Tetrahydro-alpha,alpha,5-trimethyl-5-vinylfuran-2-methanol	Floral	6	2.5	4.8	0.4	2.8	3.4	4.3	0.0	0.0
7212-44-4	Nerolidol	Floral, green, citrus	0.00025	0.0	0.0	0.0	0.0	0.0	0.0	0.0	51.2
40716-66-3	Trans-nerolidol	Slight neroli-like	0.25	0.6	0.4	0.2	1.1	1.9	0.0	0.0	0.0
78-70-6	Linalool	Floral, sweet	0.005	4227.6	7196.1	2803.1	3508.8	5112.0	7535.1	73.2	107.6
106-70-7	Methyl hexanoate	Sweet, balsamic, creamy	0.075	1.6	1.2	0.2	3.0	2.4	2.1	0.1	0.1
110-93-0	6-Methyl-5-hepten-2-one	Green, grassy, fresh	0.1	5.5	5.1	1.5	3.0	5.7	7.2	0.0	0.0
119-36-8	Methyl salicylate	Fresh, sweet	0.06	119.7	91.3	41.0	107.4	82.8	73.9	2.3	1.6
488-10-8	Jasmone	Floral	0.007	36.2	33.3	11.4	72.8	227.7	79.1	45.0	31.8
106–24-1	Geraniol	Rose-like, sweet	0.0075	14860.0	16259.5	6189.3	10919.7	13427.5	14939.0	278.3	206.7
3796-70-1	Geranylacetone	Floral, green, fruity	0.1	1.7	0.0	0.0	0.0	0.0	0.0	0.0	0.0
928-96-1	Leaf alcohol	Grass, green, fruit	0.4	1.0	1.4	0.0	0.9	0.0	1.0	0.0	0.0
590-86-3	3-methylbutanal	Malt	0.1	0.3	0.4	0.0	0.6	0.7	1.9	0.2	0.3
111-70-6	1-Heptanol	Green, sweet	0.2	0.7	1.4	0.3	1.1	1.3	1.4	0.0	0.0
111-71-7	Heptaldehyde	Green, oily, grassy	0.01	18.5	13.6	4.3	12.3	18.9	18.8	1.6	2.4
124-19-6	1-Nonanal	Floral, fatty, green	0.04	13.9	22.8	6.9	14.7	29.3	31.7	6.9	3.2
111-87-5	1-Octanol	Green, citrus, fatty	0.1	0.0	2.4	0.0	6.8	0.0	1.7	2.2	0.3
124-13-0	Octanal	Citrus, fruit, green	0.0007	96.3	49.4	0.0	79.1	0.0	73.1	6.6	14.8

It is worth noting that due to the lack of standard substances for related metabolites, relative quantification or semi-quantification is carried out by the internal standard method, which may cause a certain deviation in the accurate detection of substance content. And due to the different characteristics of compounds, this deviation effect may be different for each metabolite. Therefore, although the OAV values of many other volatile metabolites such as 2-Methylbutyraldehyde, trans-2-decen-1-ol and cis-3-hexenyl cis-3-hexenoate are less than 1, they may also contribute to the aroma of Zheng’an Bai tea (24, 50, 54, 55). However, since SPME-GC-MS/MS is a highly selective method that is very effective for aldehydes and ketones but not so effective for critical sulfur-containing compounds, we will further analyze its aroma characteristics by combining sensory evaluation methods in future studies. Meantime, when identifying differential metabolites, relatively loose standards may lead to the identification of more differential metabolites.

## 4 Conclusion

In this study, a total of 573 non-volatile metabolites were identified in total, including 96 flavonoids and their derivatives, 75 amino acids and their derivatives, 56 sugars and alcohols, 48 terpenoids and their derivatives, 46 organic acids, 44 alkaloids and their derivatives, and 39 polyphenols and their derivatives. Among the top 20 differential metabolites of fresh leaves, ethyl gallate, theaflavin, isovitexin, linalool and vincetoxicoside B are the most common. However, the differential metabolites among the samples at the processing stage change abundantly. Overall, sugars and alcohols, alkaloids and polyphenols show the greatest differences between fresh leaves and samples at the processing stage. Meanwhile, we identified 97 volatile metabolites including alcohols, aldehydes and esters. The results showed that the fixation process led to a sharp decrease in the content of most volatile substances, while 9 volatiles substances with an OAV > 1, such as geraniol, octanal, linalool, jasmone and nerolidol, were identified as the key aroma components of Zheng’an Bai tea. Due to the lack of accurate quantitative data, this study may have certain limitations. However, we not only comprehensively revealed the quality characteristics of Zheng’an Bai tea, but also revealed for the first time the dynamic change trend of tea quality at different stages from fresh leaves, processing to dry tea.

## Data Availability

The datasets presented in this study can be found in online repositories. The names of the repository/repositories and accession number(s) can be found in the article/Supplementary material.

## Footnotes

^1^ https://www.genome.jp/kegg/

^2^ https://international.biocloud.net/zh/software/agriculture/list
