# Supplementary figures and images for: Widely targeted metabolomics and SPME-GC-MS analysis revealed the quality characteristics of non-volatile/volatile compounds in Zheng’an Bai tea
Source: Front Nutr. 2024 Nov 25;11:1484257. doi: 10.3389/fnut.2024.1484257 (PMC11625558; doi:10.3389/fnut.2024.1484257)

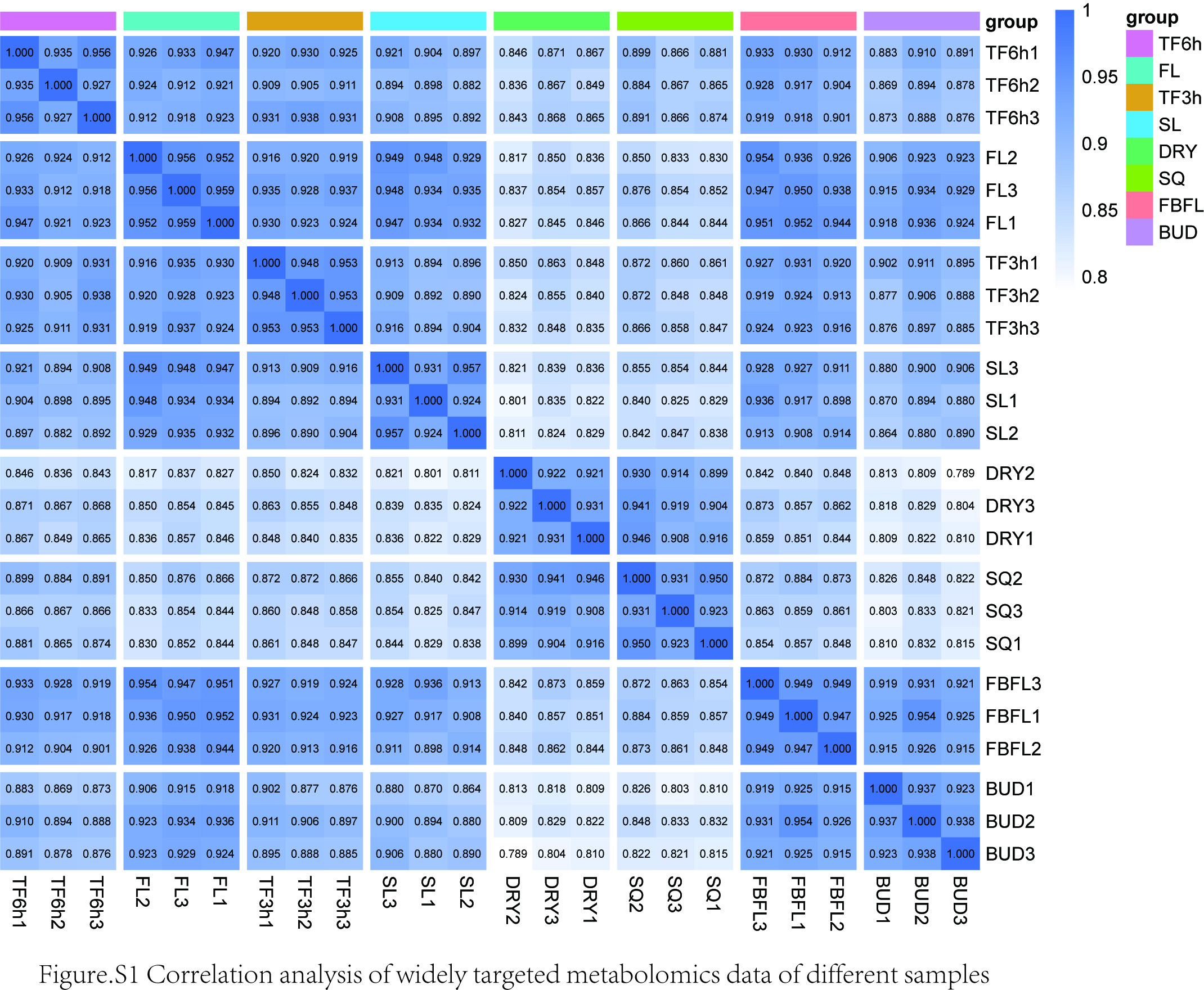

Supplement: Supplementary file 1 [file Image_1.tif]

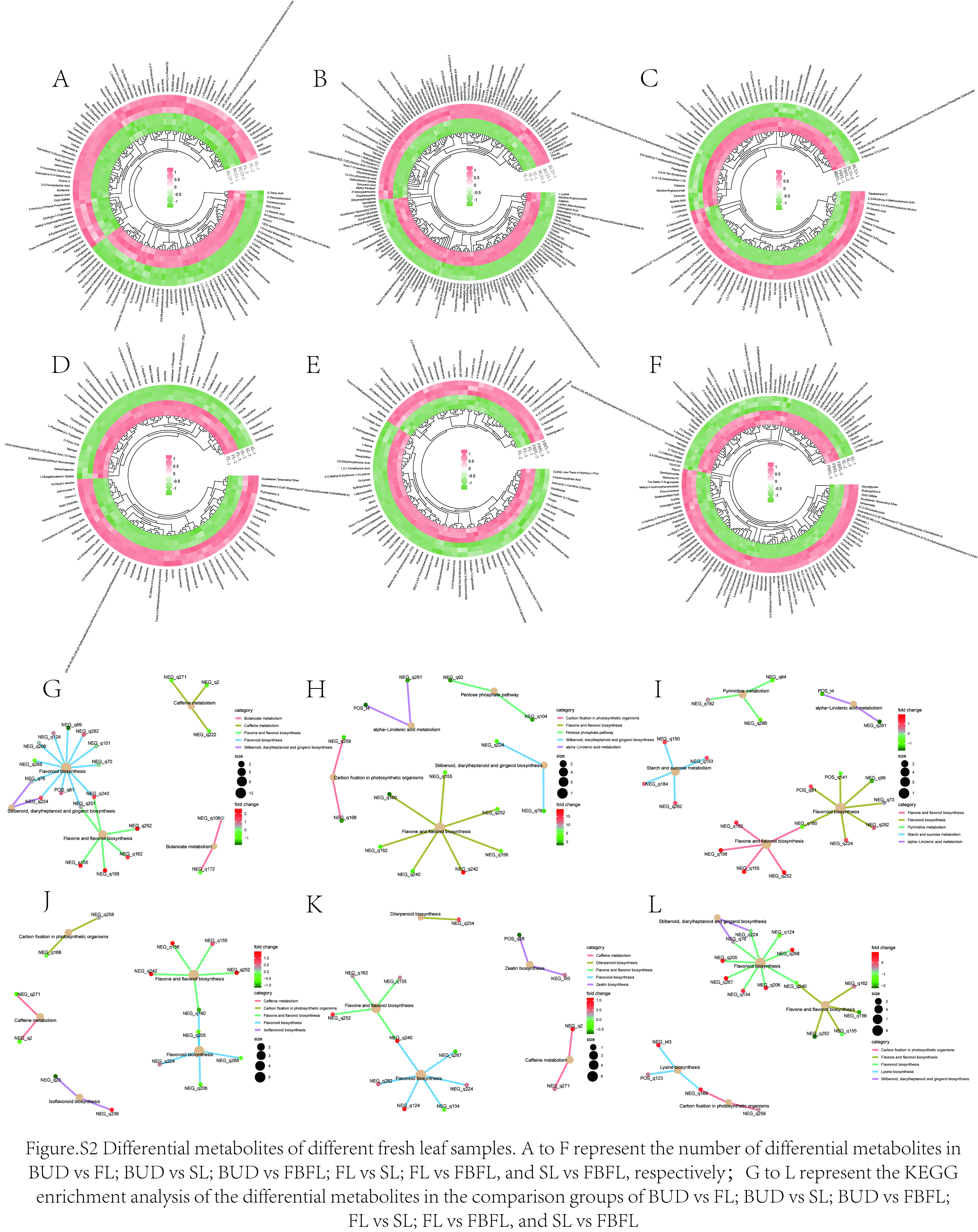

Supplement: Supplementary file 2 [file Image_2.tif]

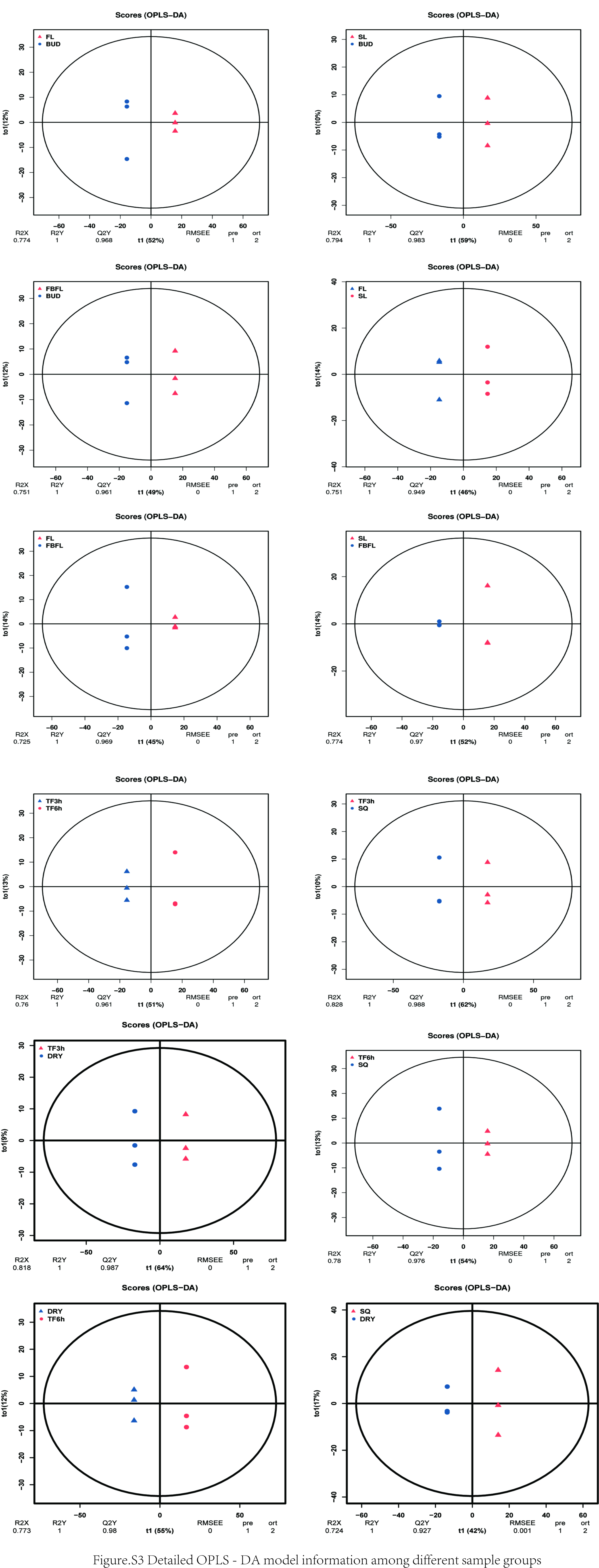

Supplement: Supplementary file 3 [file Image_3.tif]

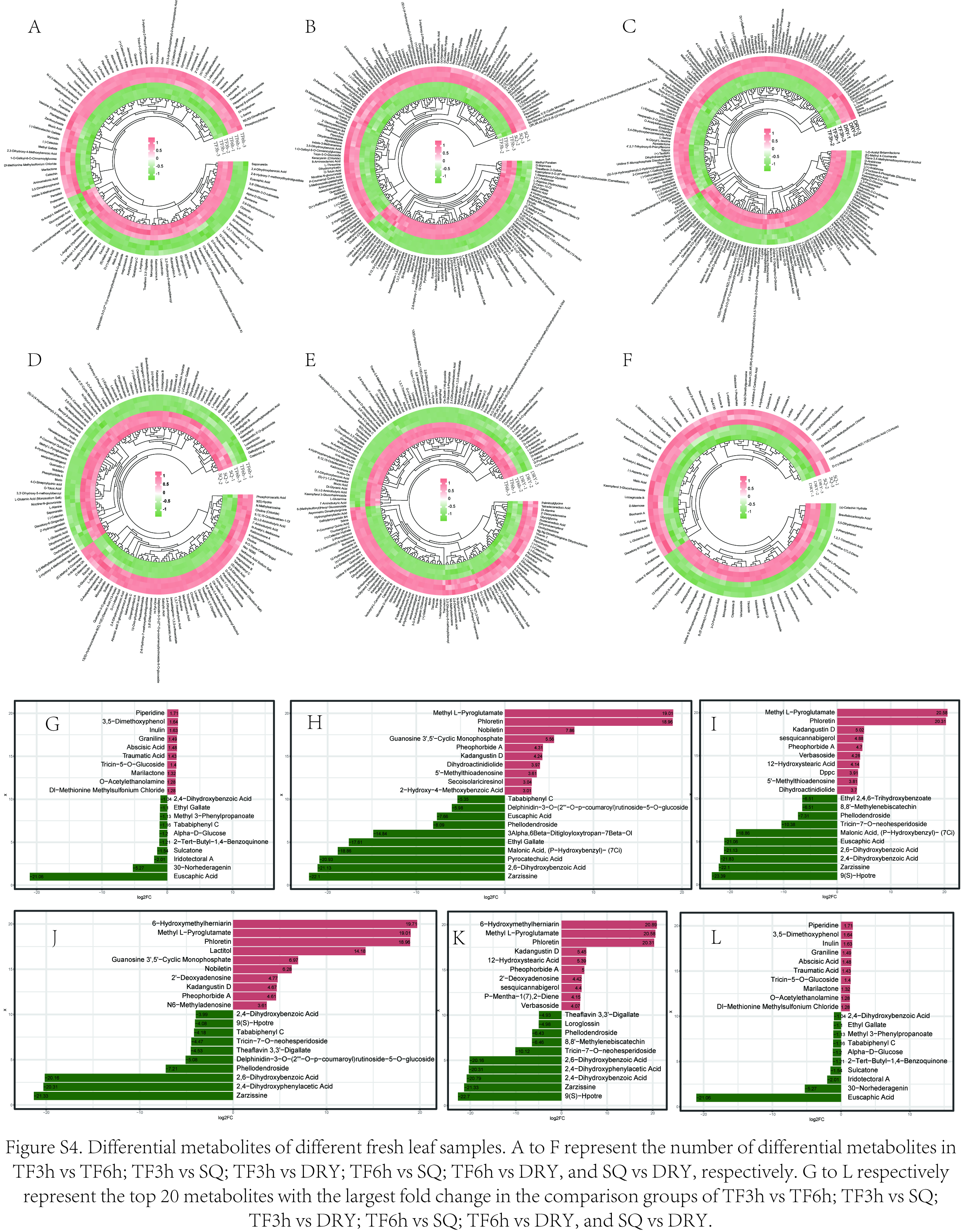

Supplement: Supplementary file 4 [file Image_4.tif]
